# Dr. Sigmond on Hallucinations

**Published:** 1848-10-01

**Authors:** George Sigmond

**Affiliations:** PROFESSOR OF MATERIA MEDICA, ETC.


### ON HALLUCINATIONS.

BY GEORGE SIGMOND, M.D., PROFESSOR OF MATERIA MEDICA, ETC.

The subject of hallucinations has excited in the French School of Psychological Medicine deep interest, in consequence of the Academy of Medicine having, in the year 1844, offered one of its annual prizes for the best dissertation on the causes which produce them, and the diseases which they characterize. There were no less than eleven candidates in the field, and it is understood that amongst them were some of the most distinguished psychologists of the day. The reward, however, was assigned to Dr. Baillarger, the physician to the Salpétrière, to whom science is already deeply indebted for his inquiries into the anatomy, the physiology, and pathology of the nervous system, and whose numerous works on mental diseases have placed him one of the foremost men of the age. He has published the essay which gained the prize, and we have also had the results of the inquiries of Brierre de Boismont, of Moreau, and of others; still, however, a vast and most important field for observation remains from which to glean results of the most important character. The distinction to be drawn between hallucinations, monomania, mental delusion, and fantasia, is much insisted upon by many foreign writers. The English school seems scarcely to admit that there is sufficient ground for a decided diagnosis; and on perusing the best works written by its leaders, we generally find that they have not noticed any classification of these different states of disordered intellect, but mix them indiscriminately together. Between hallucination and monomania, there is at any rate a most striking distinction; the latter is a fixed idea, predominating over every faculty of the mind, absorbing all other ideas, and generally influencing the actions of the body; whilst the other is a false impression, made on the sensorial apparatus, often very slightly interfering with the intellectual powers, and unaccompanied by irresistible impulse. With regard to the actual definition of hallucination and its diagnosis between mental delusions and fantasias, until we have more maturely reflected on their nature, and studied them under all their various phases, it would be premature to attempt such axioms as would be acknowledged to be both philosophically and morally undeniable. Who, after studying with unwearied perseverance the structure of the human mind, or having read the most acutely reasoned disquisitions, can fancy that he has sounded its depths, or has even pierced beyond the surface? Still do we feel that it is not inscrutable; but that, guided by reason, by humanity, and by confidence in a Supreme Power, we shall gradually have some of the beauteous truths of nature revealed to us. There is apparently a link between hallucination and extreme credulity, which has as yet not been investigated. Those who have so completely yielded up their conviction and their reason to the belief in apparitions, in demons, and in sorcery, as to have been convinced not only that there is supernatural agency constantly at work, but that they themselves have been acted upon, have had neither false perceptions, nor any condition of the sensorial apparatus that would lead to the idea that they were of unsound mind, yet have they been considered as labouring under hallucinations by the incredulous portions of society; whilst they may have been ranked according to the people amongst whom they have lived as saints, as enthusiasts, or as philosophers. They have not been men of inferior intellect, but of high and commanding capacity, who have thus been bordering on hallucinations. Volumes of great learning exist on the Nymph Egeria, of Numa Pompilius, of the Demon of Socrates, the vision of Brutus, the inspirer of Mahomet, and the mystic messengers of one-half of the saints of the catholic church; and while some of those have commented upon them as men of high character and endowments, others have characterized them as visionary dreamers, half impostors, half self-deceivers. There is scarcely a man of eminence who has written his autobiography, or laid open the secrets of his inmost soul, but has not acknowledged some preternatural event in his life; the most sceptical have felt, at some period or other, a mental emotion, either a fantasia or an hallucination. The man who, in the age just passing away, has been considered at once the most philosophic, the most worldly, and the most wedded to material doctrines, the Prince Talleyrand, never could speak of one circumstance of his life without shuddering, and without betraying an emotion, which amounted to something like an exhibition of momentary excitement, and that of the most extraordinary kind, in an individual who was remarkable for his passive bearing under every circumstance of life. The anecdote has been related by M. Colmache, his private secretary, as he learnt it from him; and his widow has assured me that the subject was always one to which, when allusion was made, the Prince displayed a most unusual state of mind. "I remember," he said, "upon one occasion, having been gifted for one single moment with an unknown and nameless power. I know not to this moment whence it came; it has never once returned; and yet, upon that one occasion, it saved my life. Without that sudden and mysterious inspiration, I should not have been here to tell my tale. I had freighted a ship in concert with my friend, Beaumetz. He was a good fellow, Beaumetz, with whom I had ever lived on the most intimate terms; and in those stormy times, when it needed not only friendship to bind men together, but almost godlike courage to show that friendship. I could not but prize most highly all his bold and loyal demonstrations of kindness and attachment to me. I had not a single reason to doubt his friendship. On the contrary, he had given me, on several occasions most positive proof of his devotion to my interest and well-being. We had fled from France; we had arrived at New York together; and we had lived in perfect harmony during our stay there. So, after having resolved upon improving the little money that was left by speculation, it was, still in partnership and together, that we freighted a small vessel for India,—trusting to all the goodly chances which had befriended us in our escape from danger and from death, to venture once more conjointly to brave the storms and perils of a yet longer and more adventurous voyage. Everything was embarked for our departure—bills were all paid, and farewells all taken—and we were waiting for a fair wind with most eager expectation,—being prepared to embark at any hour of the day or night, in obedience to the warning of the captain. This state of uncertainty seemed to irritate the temper of poor Beaumetz to an extraordinary degree; and unable to remain quietly at home, he hurried to and from the city with an eager, restless activity, which at times excited my astonishment; for he had ever been remarkable for great calmness and placidity of temper. One day, he entered our lodging, evidently labouring under great excitement, although commanding himself to appear calm. I was engaged at the moment writing letters to Europe; and, looking over my shoulder, he said, with forced gaiety, 'What need to waste time in penning those letters?—they will never reach their destination. Come with me, and let us take a turn on the Battery; perhaps the wind may be chopping round; we may be nearer our departure than we imagine.' The day was very fine, although the wind was blowing hard, and I suffered myself to be persuaded. Beaumetz, I remembered afterwards, displayed an unusual officiousness in aiding me to close my desk and put away my papers, handing me, with hurried eagerness, my hat and cane, and doing other services to quicken my departure, which at the time I attributed to the restless desire for change, the love of activity with which he seems to have been devoured during the whole period of our delay. We walked through the crowded streets to the Battery. He had seized my arm and hurried me along, seemingly in eager haste to advance. When we had arrived on the broad esplanade—the glory then, as now, of New York—Beaumetz quickened his step still more, until we arrived close to the water's edge. He talked loud and quickly, admiring in energetic terms the beauty of the scenery, the Brocklyn heights, the shady groves of the island, the ships riding at anchor, and the busy scene on the peopled wharf—when suddenly he paused in his mad, incoherent discourse; for I had freed my arm from his grasp, and stood immoveable before him. Staying his wild and rapid steps, I fixed my eye upon his face. He turned aside, cowed and dismayed. 'Beaumetz,' I shouted, 'you mean to murder me: you intend to throw me from the height into the sea below. Deny it, monster, if you can.' The maniac stared at me for a moment; but I took especial care not to avert my gaze from his countenance, and he quailed beneath it. He stammered a few incoherent words, and strove to pass me; but I barred his passage with extended arms. He looked vacantly right and left, and then flung himself upon my neck, and burst into tears. ''Tis true—'tis true, my friend! the thought has haunted me day and night, like a flash from the lurid fire of hell. It was for this I brought you here. Look! you stand within a foot of the edge of the parapet: in another instant the work would have been done.'

The demon had left him; his eye was unsettled, and the white foam stood in bubbles on his parched lips; but he was no longer tossed by the same mad excitement under which he had been labouring, for he suffered me to lead him home without a single word. A few days' repose, bleeding, abstinence, completely restored him to his former self; and, what is most extraordinary, the circumstance was never mentioned between us. MY FATE was at work. It was during those few days of watching by the bedside of poor Beaumetz, that I received the letters from France which announced to me the revocation of the decree which had sent me a wanderer to America, and I was invited to return with all speed. I could not resist the appeal, and at once decided on leaving Beaumetz to prosecute our speculation alone, and on returning to Paris immediately. Prince Talleyrand, to the latest hour of his existence, believed that "he was for an instant gifted with an extraordinary light, and that during a quick and vivid flash the possible and the true was revealed to a strong and powerful mind;" that upon this the whole of the destiny of his existence hinged; and that the sudden impulsive madness of another was destined to be his future greatness in life. This species of momentary excitement, which is not again repeated, but is remembered with the most vivid impression, is what is more immediately known by the name of fantasia. It differs from mental delusion from its absence of intensity, and from its not recurring; and from hallucination in the likelihood that certain coincidences and probabilities of something like truth were mingled with superstitious feelings. Hallucinations not only exist within the walls of the lunatic asylum, and amongst those who have an intellect completely disturbed, but individuals who, under the ordinary circumstances of life are fully capable of performing their duties, are subject to momentary impressions which have no foundation in reality, and which, from their occasional recurrence, lead to the belief that the mind is in an unsound state.

When a person actually insists that he sees, hears, feels phenomena inexplicable to any but himself, the question that arises is, are the senses that convey to the brain impressions in an unhealthy state, or are they in a normal condition whilst the imagination is the seat of the delirious error? Is there a physical cause, or is there a psychological disturbance? In order fully to study and to elucidate such questions, it is first necessary to examine the physiology of the senses, to ascertain how far they are capable of misleading the reason, and urging the imagination to dreams unconnected, and reveries which have no true foundation; and then to learn whether certain pathological conditions produce the same effect, such as cerebral congestions, venous retardation, following on arterial acceleration, febrile conditions, the different neuroses, changes of temperature, effects of severe cold, privation of food, indulgence in alcoholic stimuli, habits of narcotism, lesions of organs distant from the brain, and various corporeal states, which either sympathize with the nervous system, or call it into unwonted action. In fact, the history of mankind, and the experience of those who have had the opportunity of studying the ills that flesh is heir to, prove that the manifold causes which give rise to hallucinations can be referred to causes innumerable, which oftentimes escape the active curiosity and the careful watchfulness of the most enlightened.

The hallucinations of the sense of hearing are the most frequent; and, as Dr. Baillarger has most truly observed, they are also the most complicated. Amongst those who are decidedly insane this species of false perception is infinitely more common than that of any other of the senses; it is not only one voice that is heard, but many; it is not only the less instructed, but the intellectual; it is among men of great imagination and of deep learning. It haunts the mind in the form of a demon, as in the case of the imaginative Tasso, or as Satan wrangling upon divinity, as it did with the disputant Luther, or as a Deity revealing his will, with the contemplative Swedenborg. The most simple of the hallucinations is that of noises in the ear, such as sounds made during the night in the chimney. I have known an invalid complain of a perfectly sleepless night occurring for weeks, in consequence of the idea that dwelt upon her mind that some rooks were building a nest in her chimney. She had lately returned from the country, where a rookery was established. At the lapse of a few weeks she had an intermittent fever, after which the noises ceased. But the following year, on her return again from the country, she underwent a precisely similar state. I have always attributed this to an effect produced on the nervous system by an autumnal miasma. A singular case occurred in Paris, in 1831, during one of those bloody emeutes which that unfortunate city has had to witness. A female saw her husband, a workman, fall dead at her feet, struck by a ball. A month after this event she was safely delivered of a child; but the tenth day after her accouchement delirium came on. At its commencement she heard the noise of cannon, the firing of piquets, the whistling of balls. She ran into the country, hoping, in getting out of the city, to escape from the noises by which she was pursued. She was arrested and conducted to the Salpêtrière. At the end of a month she was completely restored. During ten years six similar paroxysms have taken place, and the delirium has always commenced with hallucination of sound. Always has this patient run into the country to escape from ideal discharges of cannon, from the firing of guns. Frequently, in the precipitation of her flight, she has fallen into the water; twice has she thrown herself into it to escape the horror of the sounds that remind her of the death of her husband, and recal the miseries she endured. Single voices are seldom so common as two voices, and the subject is oftentimes accompanied and caused by some emotion in the mind. Pariset, in his lectures, mentioned the case of a young girl who heard a voice constantly calling her thief, and reproaching her with the object stolen. At length, she returned the article, and the hallucination soon ceased. This, however, approaches to the state which our forefathers called conscience-stricken; and we have in our court of judicature more than one instance where a murderer has been pursued by a voice, as was Cain after his fratricide. One unfortunate maniac at the Salpêtrière heard a voice proclaiming the announcement of his death, and the punishment which, for his sins, he was destined to undergo. Some females who have led the most reproachable lives have heard voices calling them by the worst epithets. The hallucination which believes that echo is answering every word is, that the individual speaks what he thinks internally, but unconscious that he has spoken, and not recognising his own voice, he believes that another person repeats his thoughts. A distinguished nobleman, not long since deceased, who held at one time a high office in the administration of the country, was subject to an hallucination of this kind. He not only spoke to himself when alone, as those who labour under hallucinations most generally do, but even in the society of strangers, which is less usual.

An anecdote has been recorded for which, although the fact may not have occurred exactly as it is generally told, there is ample ground for believing that many circumstances not at all unlike it did actually happen. The noble lord took a friend, whom he had met at the country seat of another friend, in his carriage to London, arriving there rather late in the afternoon; he said, as he fancied to himself, "I suppose I must ask this man to dinner," but, from his mental absorption, he expressed himself aloud. His friend, not knowing that disease was the cause of this singularity, said, as if also speaking to himself, "I suppose this nobleman will ask me to dinner; if he does I shall not accept it." As may be supposed, a very unfortunate confusion of ideas sprang up in the mind of this unfortunate nobleman, from similar occurrences, and his days were terminated under corporeal restraint, so visible did his malady become.

Two voices in earnest conversation are not unfrequently heard, sometimes loudly disputing, at other times giving advice, the passions or the feelings of the individual who is subject to the hallucinations imparting the colouring to the subject: thus a lady was greatly disturbed, she was much given to the toilette, and had to listen to the voices of two men whom she had never seen, who constantly pursued her everywhere, leaving her no peace with the compliments that they continually addressed to her. Early in the morning, they were prepared with their praises and admiration of the clearness and beauty of her complexion. A case worthy the narration occurred to me about ten years ago, at the period when my lectures on the Materia Medica were printed weekly in the "Lancet." The person who conducted Mr. Wakley's printing establishment, in Essex-street in the Strand, called on me one morning, and requested my professional assistance for one of the printers immediately under his own direction, who had lately betrayed a singular species of hallucination, for which his friends were exceedingly anxious that he should obtain medical advice. He at length consented, although he believed himself to be in the most perfect health; but, as in one of my lectures I had spoken of a case which was peculiarly interesting to him, in consequence of its allusion to the appearance of imps to those labouring under delirium tremens, he would have no objection to an interview with me. He was described to me as an individual of superior ability to those of his own position, a man fond of reading, and particularly attentive to medical literature; he had lately been gloomy, misanthropic, and, in contradiction to his former habits, which were abstemious, he now passed much of his time at the public-house, and had been frequently intoxicated, but rather from a wish to drown thought than from a love of liquor. On an appointed day he presented himself to me, accompanied by a friend; he was evidently labouring under great embarrassment, and seemed unwilling for some time to place confidence in me, for I could not get any acknowledgment that he was unwell. At length, he began by imploring me to forgive the unusual request he was about to make, and to bear in mind that nothing but the great misery he endured could lead him to ask me to take the only steps that could relieve him. He then told me that he had been persecuted for several months, by the unwearied attacks of two imps which had taken up their residence in his body; their size was exceedingly small, but their voices were tremendously powerful—one was a lively, agreeable, merry devil, that had constantly something jocular to say, but the other was a most sulky, unhappy wretch, who was constantly tormenting him, and advising him to commit suicide; he in fact it was that rendered his whole life one scene of wretchedness. He had never confided his sorrows to any one but the friend that accompanied him, and he should not have done so, but that he was in some measure obliged to do it, for, whilst walking with him some days ago, in Fleet-street, he had been under the necessity of borrowing his pocket-handkerchief, a thing he was too well aware was so unusual and so ungentlemanlike, that he felt it a duty he owed to himself to explain the cause, which was, that, on leaving the house, he had placed his pocket-handkerchief in his hat, where, by a lucky accident, the two devils were at the moment, that they of course were in confinement upon his head, where he hoped to keep them; but the merry little imp had suggested to his companion that if they tickled the nose of their victim, he would be obliged to take his handkerchief out of the hat, and they would escape; to obviate this misfortune it was that he borrowed one from his friend. All this was told with the greatest gravity, and then came, with a most piteous look, the prayer of the petition he had to make to me. After the usual compliments paid to physicians for their skill and humanity, and the assertion of the belief that I was the only man in London that could restore him to health, he proposed that every aperture in the room should be carefully closed, that the key-hole should be stopped, and that not the slightest chink should be left open, whilst he stripped himself naked, and I hunted after the imps, seized and confined them. This was the only remedy for the state in which he was. I humoured him to the top of his bent, I listened with the appearance of belief to his melancholy narrative, and commenced the shutting up the crevices; I soon saw, however, how useless any attempt would prove to relieve his mind. It was evident that, however hermetically sealed the apartment, the idea was too rooted ever to be removed; and I was, after some length of time given to his assistance, compelled to conclude my interview. Of course, I could only foretell to his friends the sad termination of the case, which, I afterwards learnt, he hurried on, by his complete abandonment to the momentary respite that spirits and fermented liquors gave him.

One of the most singular hallucinations to which the sense of hearing lends itself is, to the carrying on a long uninterrupted conversation, during which the individual speaks, addresses a third party, and waits to listen to the response, which seems to be perfectly new to the apparent listener, who gives every attention. Who that reads the life of Tasso, as given by his friend and biographer, Manso, does not remember to have either himself met with a patient who has reminded him of the description, or has heard from a medical friend some tale which has carried the same marvellous air with it. Any one accustomed to what occurs within lunatic establishments must have seen patients walking up and down, holding an imaginary conversation, or must have heard, during the night, in some cell or other, an earnest long-continued dialogue. When Tasso, to convince his friend, Manso, of the folly of his incredulity, admitted him to his privacy during the visit of the familiar genius with whom he was in communication, he turned his head towards the window, ceased to reply to any observations but those which he supposed were made by this mysterious being. The language, the style he made use of, were of the most elevated character, and he waited with great composure for the reply which he believed that he heard. Manso, astonished at what passed, though he could see nothing, was held in a species of ecstasy; he gazed with wonder at the spot pointed out to him, saw only the rays of the sun shining, and felt an indescribable sensation, which was only relieved by the supposed departure of the spirit, when Tasso asked him if he were yet freed from his doubts, to which Manso replied, that they still remained, although he acknowledged he had heard extraordinary things. It has been often observed, that during the hallucinations there is a higher degree of intellectual power exhibited than the individual suffering under its influence is supposed, in his ordinary state, to possess; some of those who deliver extemporaneous discourses have been heard to pour forth, with the greatest ease, the most felicitous expressions, the most poignant satire, and the readiest wit. During the hallucinations produced by taking the Indian hemp, the intensity of the sense of sound is most striking. The celebrated Theodore Gaultier related to Dr. Moreau, in poetic language, which it is hopeless to attempt to translate, so as to give an idea of the style of this highly imaginative author, the sensations produced; he says, that "his sense of hearing was prodigiously developed. I actually heard the noise of colours—green, red, blue, yellow sounds, reached me in waves perfectly distinct; a glass overthrown, the creaking of a footstool, a word pronounced low, vibrated and shook me like peals of thunder; my own voice appeared to me so loud, that I dared not speak, for fear of shattering the walls around me, or of making me burst like an explosive shell; more than five hundred clocks sang out the hour with an harmonious silvery sound; every sonorous object sounded like the note of an harmonica or the Æolian harp—I swam or floated in an ocean of sound." Such is the exaggerated language which has been employed by an individual whose taste and enjoyment of music have rendered his criticism on that art so much sought after. Doctor Carrière, who is one of those who have made experiments with the *haschych* upon several medical pupils, employs similar terms when speaking of its effects upon the sense of hearing. One of his brother physicians was under its influence, when a servant girl in the next room began an ordinary song; he put his ear to the key-hole in an apparent ecstasy of delight, as if he were unwilling that the least sound should escape him. He remained under the charm for nearly half an hour, until the heroine of the mop and pail quitted her work. The situation from which the supposed voice emanates has not yet been studied with much attention, but there is every reason to believe, from the few remarks that have yet been made, that some light may eventually be thrown upon the subject by a more minute examination. It has been observed, that the epigastrium has been referred to as the spot from which voices are generally said to issue; it is the remark of Bertrand, that all those who profess to have the power of magnetic somnambulism say, that they derive it from a voice seated in the epigastrium, and the greater number of highly nervous persons, of epileptics, and *convulsionaire*s always refer to that spot. The case of the individual, mentioned by Pinel, who spoke of that region as the centre of every pain, grief, pleasure, or emotion, will be remembered, as affording an ample specimen of how much sensibility is said to be concentrated in the epigastrium by those who suffer under derangement in which melancholy is the principal characteristic. It will be remembered that in all ages the voice has been said to proceed from the abdomen, during the contortions of the sybil, the enchantments of the sorceress, the ravings of the false prophetess, the dreams of the somnambulist, or the ecstasies of the religious enthusiast. From the pharynx and from the chest a voice issues, more especially amongst the epileptics and the hysterical; they hear unearthly sounds, more generally of a shrill kind, occasionally howlings—they seem to inspire the same eagerness for imitation as do sounds from the epigastrium, and they depend more generally upon a heated imagination springing from the individual. The top of the head is not unfrequently described as the position from which the voice comes. When there are two voices, they are spoken of as issuing from two different parts of the head; in one case, observed by Dr. Baillarger, a man of condition had one voice at the back of his head, recommending self-destruction, another at the anterior, dissuading him from it. Occasionally the two hemispheres appear to be the seat of antagonist voices, each inspiring a different train of thought, and more generally opposing each other. There is much in these hallucinations which has for its belief the existence of two ideal beings addressing the disordered mind, that might indicate some degree of probability in the idea, so well thrown out, of the late Dr. Wigan, of the duality of the brain. Many are the cases which seem to have had considerable light thrown upon them by this theory, which remains yet to be more closely investigated before one can presume to give it denial. The observations which it has excited at the institutions in Paris, have led some pathologists and physiologists to become followers of the doctrine laid down. When they are more matured, it is to be hoped they will be given to the profession, that they may further prosecute inquiries which, in the present stage of psychology, must be considered as valuable, and as steps to a higher ground than we have yet been able to take in our pursuits.

In the consideration of the false representations made to the brain by the eye, we more immediately recognise the truth of the line of demarcation drawn by Esquirol, and followed by the succeeding French school, between hallucination and mental illusion. In the latter, there has been some impression made upon the external nervous system, but it is falsely conveyed to the brain; there has been something that has impinged upon the optic nerve, but during the transition to the brain it has acquired some undue character from the imagination, and it is represented under some new and imaginary form, whilst during an hallucination both the external and internal portion of the sensorial system are deceived, and have altogether created the object which is presented. During an illusion, a huge black cat by the fireside has been mentally transformed into the image of a beautiful woman; a white garment floating in the wind into a celestial messenger; the dark miserable alleys of Cairo have been lighted up into magnificent bazaars, and a brilliant fête has been seen where a few beggars have been assembled. Hallucinations of sight, where the fancy is altogether the creator, are very rare amongst the insane; the eye, more faithful than the ear, does not picture objects which have no reality whatever with any very great facility; when it occurs, it does not betoken a more decided state of insanity; however, there are many persons who see for a considerable length of time visionary objects who are well fitted for all the ordinary duties of life. It has been observed, that those who once have been thus misled by the senses are liable, not only to its recurrence, but to the appearance of similar deceptions. So truly does Shakspeare seem to have known the workings of the human imagination, that he paints Macbeth as yielding to two hallucinations—first, the air-drawn dagger, "the false creation proceeding from the heat-oppressed brain," then the "strange infirmity, the very painting of his fear," Banquo, "the horrible shadow," the unreal mockery; he describes Hamlet as twice beholding the ghost of his father, and yet capable of fulfilling the ordinary duties of life, whilst Lear, in the most incoherent ravings of mania, is beset with no visions. The illusions produced by the sight are common. The whole history of ghosts, of preternatural appearance, of special wonders, must be deeply studied before an attempt can be made to elucidate them; and, in the present infancy of psychological science, however large the data may be, we want several links of the chain; these, however, will doubtless be gradually furnished to us as enlightened medical men study the phenomena, and bring their labours, however slight they may be, into the general fund of knowledge.

The imagination is engaged in a very different manner where the sight is in fault than where hearing is disordered; it does not paint such exciting scenes, it does not bring the reason into action, as we have seen it do during the disturbance of the latter organ, when conversations sometimes of an intellectual character occur, where the individual has to listen to the advice, the reproaches, or the menaces of a supposed stranger. It is generally one object alone that attracts the attention, or that is complained of; it may appear under various shapes, but it is more generally connected with some idea that has previously struck with great intensity on the mind; thus the learned and ingenious Pascal, after being in danger of his life at the bridge of Neuilly, saw afterwards a precipice with a fearful abyss at his feet. Those whose minds are strongly bent on devotion, and have yielded up their thoughts to religion, see angels and the Virgin Mary. I was once in a church where the clergyman, who had for some time betrayed symptoms bordering on alienation of mind, but who never had evinced its actual presence, broke off his discourse, pointing to the presence of the Holy Ghost; he was fortunately prevented, by timely attention, from becoming insane, but he was considered ever afterwards incapable of resuming his duties. It is generally known that Napoleon Buonaparte was, in the early part of his career, subject to an hallucination of sight, in consequence of the vivid impression made upon his mind by one of the occurrences of his eventful life. In the heat of one of the many battles in which he was engaged, he was carried, by the ardour of his courage, into the very midst of the slaughter, his immediate followers fled, he was left alone, surrounded on all sides by fierce assailants; how he escaped from death unhurt no one was ever able to ascertain—it was one of those miracles which seemed to be worked by his tutelary genius; the deep impression, however, of the danger which he had run was not effaced when he mounted the throne; at certain intervals, a striking hallucination occurred—suddenly, in the midst of the silence of the palace, loud cries were occasionally heard, the emperor was seen fighting with the utmost desperation amongst his visionary foes; it lasted but a very short period, but during that time the battle seemed to be a tremendous one. This gave rise to the report that this great general was subject to epileptic fits; but the fact, as I have stated it, was commented upon by Pariset in his lectures.

These visions will not unfrequently cease upon shutting the eyes; they more generally, however, are permanent. Esquirol used to state that he was called upon to attend one of the relatives of Napoleon's family who had been in the army: after several reverses of fortune, he exhibited undoubted signs of insanity, and was placed under the immediate care of the great physician, who has left a name more respected and admired than that of the mighty emperor, who patronised the humble superintendent of a lunatic asylum. His were both delusions and hallucinations, for he saw around him members of the imperial family; and when the servants attached to the establishment did not pay due homage to these great personages, his anger knew no bounds, and he worked himself up to a high state of irritation. He fell upon his knees before one of the imaginary beings, and implored protection and pardon. In the midst of one of the most outrageous of his paroxysms, Esquirol advised him to place a bandage over his eyes. To this he consented: instantaneously, all his delusions ceased; he no longer saw any imaginary beings; there was nothing to excite him any longer; he became perfectly calm and collected, and even spoke rationally of his state of mind. The experiment was afterwards frequently tried, and was always attended with the most complete success. On one occasion the bandage was kept on for twelve hours, during which he was completely rational, and did not betray for one moment during the whole of the time the slightest appearance of his malady; but at the end of that period, on his eyes being relieved from that state of darkness, the delusion recommenced.

Sometimes a train of different ideas arise; the hallucination is complex; the removal of one visionary object is followed by the presence of another. Dr. Moreau relates a case that struck him very forcibly when he entered as a pupil at Charenton, and which has subsequently from circumstances occupied his attention. A young lady, a near relative, of lively imagination, had been subjected to some chagrin which preyed much upon her spirits, but which produced no bad effect either upon her health of body, or upon the general functions of her mind. Returning home with a younger sister in the evening, she had scarcely put her foot upon the first step of the staircase which led to her room, than she fancied that the whole of the staircase was enveloped in flame. Being a person of great presence of mind, she reflected that there could be no reality, but that she must be the sport of her fancy. She, therefore, courageously went into her room, knowing where some allumettes were kept, she felt for them in the dark, but being naturally in a state of great agitation, she threw the box that contained them on the ground. On stooping to pick them up, she uttered a most fearful cry, which excited general alarm: she had seen extended at her feet the dead body of a man. Her sister, who was not in the slightest degree aware of the cause of her alarm, immediately seized one of the phosphorus matches, and with its light the whole hallucination disappeared, never to return again, but it left behind an indelible impression. Dr. Moreau has given us the result of his investigation of the case, and his attempt to connect it with some previous occurrence, and with either hereditary or constitutional predisposition to some unusual psychological state. Salverte relates an instance where a female was bitterly bewailing the loss of a brother, when suddenly she heard his voice, which by an unwarrantable deception, somebody near her imitated in such a manner as to deceive her. Overwhelmed by fright, she exclaimed, and firmly believed, that she saw his shade surrounded by a resplendent halo of light.

If one gave way to the belief in the numerous tales that are afloat upon what is called indisputable authority, of hallucinations having been succeeded by the actual occurrence of what presented itself in the dream, one would be lost in a labyrinth from which it would be impossible to escape; besides which, such is the credulity with which some of the wisest and best of men are endued, that they unintentionally lend themselves to the propagation of marvels which do not bear the test of slow and careful inquiry.

Some individuals do not see a single object; they have represented before their eyes different objects, generally, however, bearing upon one matter that absorbs their thoughts. A lady who had been one of the most decided devotees to the card table, became impressed with the hallucination that she had succeeded to a large fortune, and that it was necessary to exhibit great hospitality. She fancied two evenings in the week that she received her friends, laid out the card-table with great exactitude at the proper hour; went through all the courtesies of the hostess with great tact and politeness, looked over the hands of visionary partners; praised their skill at the game; hoped they would reach home in safety when she took leave of them, and acted with precisely the same politeness that she would have done had she not wandered in her imagination. Bonnet in "*L'Essai Analytique sur l'Ame,*" tells us that he was acquainted with a man full of health, of honesty of judgment, and of merit, who in open day, and independently of all external impression, saw from time to time before him figures of men, of women, of birds, of buildings, &c. He saw these figures make different movements, approach, retire, go away, diminish, increase, appear, disappear. He saw buildings rise up before him, and exhibit all the different parts that enter into their construction; the carpets of the apartments change into richer carpets: sometimes he saw rich tapestry on the walls, on which hung pictures resembling beautiful views in the country; then quickly all this would give place to less ornamented decoration, or to common materials. All the paintings seemed to be of the most perfect beauty, and to represent the objects for which they were intended, with the same life as they did naturally; but these were only paintings, and neither the men nor the women ever spoke, they were perfectly silent; nor was the slightest noise attendant upon anything that he saw. This person had undergone for both eyes the operation for cataract at an age somewhat advanced. The great success which at first followed the operation would have continued to be permanent, had he taken proper care. What is most remarkable is, that this individual did not, like visionaries generally, take these visions for realities; he judged perfectly correctly of their want of reality. These apparitions are to him, that which his reason knows them to be, and he looks upon them as a source of amusement. He knows not at what moment a new vision may present itself, and under what form it may come. "His brain," says Bonnet, "is as a theatre in which the scenery is executed by machines, which afford the greater pleasure to the spectator because they are totally unforeseen and unexpected."

Where the mind is completely taken up by a particular thought, and bodily disease (more especially of the nervous system) supervenes, there are innumerable instances of visions: some of them, if by a strange coincidence they had been borne out by the actual occurrence of some singular events, they would have passed into the regions which the lovers of the preternatural love to explore; thus, the Marquis de Rambouillet, the eldest brother of the Duchess of Montansier, was in conversation with a young friend, the Marquis de Precy—they were neither of them much more than twenty-five years of age, and like the young nobility in France, were destined for the army, and when about to join their regiments they discussed together the events of this life, and the probability of a future state. As they both seemed rather of a sceptical turn of mind, they doubted whether, on the occurrence of death, the soul might not, before leaving this earth for ever, visit a friend; and each promised the other, that in case of their falling victims during the battle, the one dying should visit his surviving companion, and make him acquainted with the nature and causes of his death. At the end of three months, the Marquis de Rambouillet went to Belgium, where the war had broken out. De Precy was seized with a violent fever, which compelled him, in spite of his wishes to join his regiment, to remain altogether in Paris. Six weeks after, when De Precy became convalescent, he heard, as he was lying on his bed, about five o'clock in the morning, some one draw the curtain bed, and on turning round to see from whence the noise came, to his great astonishment he saw the Marquis de Rambouillet booted and spurred. He jumped out of bed immediately, and rushed to throw his arms round his neck, to testify the pleasure he enjoyed at his safe return, but Rambouillet, retreating several steps, repulsed him, and said that his reception was altogether out of season—that he had only come there with a view of fulfilling the promise that he had made—that he had been killed the previous day in the trenches—that all that had been said of another world was true—that he ought to think of living in a different manner—that he had no time to lose, for that he would be killed the first opportunity that presented itself. The astonishment of the Marquis de Precy at the language thus held may be well understood. Not believing what he heard, he made still further attempts to throw himself upon the neck of his friend, whom he imagined to be laughing at him. His efforts were in vain; he found that he was embracing the air, and Rambouillet, pitying his incredulity, exhibited to him the wound which he had received; it was in the region of the kidneys, and blood seemed still to flow from it. After this, the phantom disappeared, leaving De Precy a prey to fright easier to comprehend than to describe. He repeated the tale to the whole house. Had, by some coincidence, the Marquis de Rambouillet actually been killed about this period, what endless versions of the marvellous appearance of the young Marquis would have circulated; as it was, everybody attributed the vision to the access of fever which acted upon his imagination. There are, however, so many instances of visions appearing to persons labouring under febrile excitement, that they can scarcely form an object of the inquiry; they are observed at all periods of fever, sometimes during the incubation, at others during the crisis, frequently at the decline, and occasionally when convalescence has been pronounced.

It has been said that there are many states of the body which predispose to the visions of hallucination, enormous abstraction of blood, from emptying the minute vessels of the eye, has given rise to an altered condition of the optic nerve, during which strange effects have been produced upon the visual rays; starvation has the same effect; the narratives of ships perishing at sea abound with singular phenomena; the famished victims have seen not only beings before them luring them on with promised food, but they have had painted before them the most beautiful scenes which the imagination can display, gardens abounding with Hesperian fruit, crystal streams, delicious rills, ever blooming flowers, and all the fascinations that the poet and the painter give to the Elysian fields; sometimes, angels minister to them, robed in celestial garbs, and their last hours are rendered happy by the delusions to which the senses gladly lend themselves. There are certain tonics which also have an effect somewhat extraordinary; of this nature the preparations of iron more especially: this is a subject which requires some investigation, the authorities on which the knowledge of the influence of this mineral rests are very vague; sundry hints only are thrown out. My valued friend, Dr. Moreau, in that charming volume which contains his *Etudes Psychologique*, says: "On a dit et on sait, certains principes toniques excitateurs, tels que le fer, peuvent donner lieu aux mêmes accidents nerveux, aux mêmes anomalies intellectuelles;" and in a similar way do several authors speak of the effects of iron, more especially when administered to delicate females, but neither in my own practice nor in that of the many friends with whom I have communicated, have I learned any particulars springing from actual experience. The preparations of iron have, from the numerous compounds formed with it, resumed their reign in a vast number of cases in which quinine had been preferred, in consequence of this remedy producing some singular cerebral affections, determining, as it does, to the head, and causing, if not disorder of the senses, an undue stimulus to the vessels that supply the brain. Congestions have so frequently followed its use, even in ordinary doses, that it does not maintain its character. It will, therefore, be of importance to ascertain whether hallucinations have been observed after the use of iron medicinally, and whether, on the principle now generally received in mental pathology, (that which will produce a symptom will cure it, the grounds upon which hyoscyamus is now administered in maniacal excitement, and belladonna in excess of emotion), it may not be introduced with hopes of success to relieve the disordered fancy from some of those hallucinations which it is imagined to cause.

Hallucinations affecting the sense of smell are not unfrequent; but they seldom attract much attention, and unless they exist in unison with some more striking derangement of the sensorial system, afford but little scope for observation. It is very generally associated with a deranged state of the sense of taste; but this does not necessarily occur. An insane person believed firmly that he could detect the existence of cholera by the odour which followed it everywhere. He was first struck with it, he said, whilst dining; it came upon him like the smell of a dead body: he recognised its existence at Orleans, and at Bourdeaux, directly he entered those cities.

Esquirol had under his care a female, who fancied that she had a most disagreable odour about her; and on being asked to go into the garden, refused, under the plea that she was well aware that she should kill all the vegetables there by the scent that she bore. The late amiable, learned, yet eccentric Thomas Taylor, who translated the works of Plato and the Greek philosophers, who was an enthusiastic believer in the heathen mythology, worshipping Jupiter, Venus, and Mars, and on one occasion complaining that his landlady had turned him out of the house, because he wished to sacrifice a bull in her parlour to one of his gods,—insisted upon it that his wife exhaled at all times a most ambrosial smell, which captivated all who approached her. I have heard him dwell with rapture on the fact, and I was led to ask its confirmation from one of his intimate friends, who remembered her; but so far from bearing witness to the fact, he assured me that she was anything but attractive in her habits of cleanliness. I have known patients who believed that every object round them was impregnated with some disagreeable odour. This is not at all uncommon towards the termination of fever, especially if any sordes have been allowed to accumulate. Some individuals have had an idea that their breath would infect any one that approached them, and have carefully driven away nurses and young children from them, lest they might be the unconscious instrument of mischief.

Some persons, before death from phthisis pulmonalis, complain of the smell of charcoal, and declare that every object that is brought before them is impregnated with it. This, however, may depend in some measure upon the odour exhaled from the vomicæ in the lungs, or from the generally vitiated secretions.

Those who enjoy religious ecstasies among maniacs, speak of the delicious perfumes, of the divine exhalations, of the camphor, the myrrh, the frankincense—the food is holy manna, and the blood is that of the lamb, sweet yet savory. The language used by these poor beings is generally that of happiness, and they are frequently made partakers of some delicious repasts, which ordinary mortals know not of. Happy indeed are they who thus soothe the sad affliction which hangs over them, and give to their relations and friends so much sorrow and anxiety.

The hallucination of touch varies exceedingly. It is singular enough to find, in an establishment where an individual has been admitted who believes that he has rats crawling over him, that spiders infest him, that he receives occasional blows from an unknown hand, how very soon several others of the confined persons take up the same notion; and if by any chance suspicion falls upon an attendant that he has been accessory to a blow, all the others who complain, whether from cunning, or from a wish to obtain the compassion which is generally shown, load the servant with charges of being the person who annoys them. Some invalids will insist upon it that cold water has been thrown on their heads; others that corrosive substances, poisonous powders, have been thrown upon them,—that hence their bodies are metamorphosed,—that they are unlike what they once were,—and that they are grossly maltreated. Some of them cannot bear the slightest breath of air to blow upon the body: those who have witnessed the horror expressed by patients labouring under hydrophobia when the least air falls upon them, can judge of the horror which some experience when they fancy that they are blown upon.

A decayed actress who had become melancholy, after expatiating with considerable energy upon the miseries which were inflicted upon her by unknown hands, added, "They are not satisfied with these cruelties, but they are employed blowing, night and day, upon my skin, which is as pure and as unsullied as my heart, ingredients which destroy me."

One poor fellow, upon whom the trial of Madame Laffarge had produced such an effect that he believed his wife was following her example, and, wanting to get rid of him, was slowly poisoning him, would not get into bed, in consequence of her throwing poisoned powder in it, and was thus kept in a state of the most tremendous agitation. He took upon himself the precaution of locking up his clothes, and hiding the key. Soon, however, he discovered that his supposed wicked wife disseminated powders in the air; he respired nothing but poison; he could no longer bear the horrors which accumulated around him: at last, in a paroxysm of violent madness, he struck his wife repeatedly with a hammer, left her for dead, and then inflicted upon himself several severe wounds, from which he eventually recovered, but his mind is completely lost. Many patients believe that they have swallowed animals, reptiles, insects; and even those who have no other indication of the slightest alteration of intellect, cannot be induced to lay aside the impression. Sometimes they beat the stomach and bowels with great violence, often wounding and severely hurting themselves. They assert that the internal organs have disappeared; they know it by the sense of emptiness, by the hollowness of sound; they occasionally accuse a friend of being the cause, or they lay it at the door of some one to whom they have taken, without apparent cause, a violent aversion. Spiders and mice are frequently charged with being the cause of the mischief, and of having entered into the stomach. Sometimes the head is very light, at others it is enormously heavy; sometimes one arm is longer than another; there may be three arms—in fact, when the sense of touch and the general sensibility are disordered, hallucination appears in a thousand indescribable forms, wearing a Protean shape, altering every day, and exhibiting itself under the most extravagant guises. Sleep vanishes under their influence; day and night, for a series of years, is the unfortunate individual haunted and persecuted; devils take them by the feet during the night, strike them constantly upon the back at the moment they most require repose; they are seized by vampires, who, during the night, suck the blood from their veins, till atrophy and deformity of their organs takes place. Invisible agency is constantly at work.

There are cases—especially in diseased states, such as delirium tremens —in which the senses all partake of the hallucination alike; the eye, the touch, the hearing, the smell, and the taste, are so disordered, as to convey unhealthy impressions to the brain. Most generally one of the organs so predominates over the other, that its deviations only are complained of; it is only by examination of the invalid, and by repeated conversation, however, that this is perceptible. At the Salpêtrière there is at present a female, about sixty-five years of age, who has now been five or six years of unsound mind; she makes daily complaints of the frightful sufferings she has to endure, and which are consequent upon the hallucinations in which all her senses are wrapt. At night she sees forms that menace her,—heads of bodies which frighten her. Sometimes it is her own image, her own portrait, that is represented to her. Once she saw her mother, who has been some time dead, crawl towards her on her four paws. She constantly hears voices which insult her; oftentimes they tell her melancholy tales,—for instance, they repeat that her mother is dead. They send the bodies of putrefying children to her. She has sometimes the complete odour of arsenic. This woman will eat nothing but bread, because both flesh and vegetables taste of arsenic. Besides all this, she receives blows upon the head—upon the limbs. They give her cramps in the legs, icy sweats, colds; they take away her breath, and drive the blood to her head.

Having thus briefly illustrated the opinions that have issued from the French school on the share taken in the senses in the production of hallucination, the next question to be discussed is, the phenomena exhibited by the internal faculties,—such as the involuntary suspension of memory, the excitement of the imagination, and the general alteration that occurs in the reflective powers. The psycho-sensorial study carries with it the deepest interest, and demands the most profound investigation. Difficult as is the task, I trust that in your future pages you will allow me to pursue this subject; trusting that it may induce others who have had the opportunity of acquiring knowledge to aid me in a research most interesting, yet, from circumstances, most difficult—one which every one must feel to be of vast importance; and however limited may be the power of enlightening others, yet he who gives that little only makes a slight return to the profession from which he has acquired all the medical information that he has gained.

As far as examination after death has, however, enabled us to draw our inferences, we find that the effects produced by those diseases affecting the brain, are exhibited in the membranes that cover it, and upon its superficial surface, rather than upon the substance of the organ itself, for, the vascular system being for the greater part exterior, and in that system the lesions that are more remarkable taking place, it results that the inflammatory diseases, the flow of blood to the arteries, the retardation in the veins, and serous effusions, are visible there. But neither the thickening of the arachnoid membrane, granulations on the surface of the ventricular cavities, redness, nor suffusions, nor any sort of vascularity, form destructive anatomical characters by which we can arrive at any undeniable conclusions as to the pathological condition which has existed. We find all these states consequent upon congestion produced in the last moments of life, either by convulsive epilepsy, by apoplexy, by embarrassments of breathing, of circulation, by agony long endured, by long-continued cephalalgia, by fever. The deeper-seated lesions, the scirrhus, the tubercle, the softening of the brain, the cyst, are discoverable in diseases producing another train of phenomena. When we trace the paroxysm which produces violence analogical with any of the diseases of the brain known to us, we should point out meningitis as that to which it most approximates. There are many symptoms in common with it which might lead to the idea that they had some common origin. There is the same sudden outbreak after a state of quiescence and apparent stupidity. A perusal of the works of Abercromby, of Lallemand, of Bouillaud, of Lelot, and an attentive deduction from the remarks which each has made, can, however, alone furnish us with data upon which to enter on the discussion, and this would demand far greater space than could be allotted here. It would, however, be desirable to ascertain what the morbid examination in lunatic asylums can furnish us with, and unfortunately we have not had much publicity given to those investigations. The theory that our limited anatomical knowledge at present allows us to construct, is, that the debilitating passions have a retarding influence on the secretions, upon the circulation, and the performance of the functions of the organs of the human body; the most depressing of these is fear. In the disease to which we have directed attention, this seems in the first instance to attack the patient; he has a dread of the most overwhelming kind, which gradually grows into a complete oppression, weighing down all the other faculties of the mind; it is utterly in vain that he attempts to drive away the fixed idea; it remains rooted to the very inmost part of his being; the very circumstance of his attempting to drive it from him only roots it still deeper; at length, completely overwhelmed with the intensity of the suffering, he yields himself up to that sole persecuting thought; there is a complete personal inertia which forbids him further to mingle with the world, the consciousness of surrounding objects is lost, as well as that internal conscience which governs the moral actions of man. The result of this state of the mind is quickly conveyed to the body, which not only sympathizes with it, but is governed by it; it is not only that the appetite fails, the power of enjoyment, and the desire for locomotion, but the nutrition of the body ceases, the secretions are not duly performed, the circulation becomes languid, and all the organs are in a state of torpor; this proceeds, gradually increasing, till there is almost a suspension of all the powers by which life is carried on; the blood, no longer oxygenized, is replete with carbon, it slowly meanders through the liver, where the veins, congested with the material not duly carried from it, begin to stagnate with their fluid; the brain is, after some time, surcharged with this venous blood, and that state which approaches the third stage of intoxication, where there is decided pressure upon the brain, exists, the result of the arterial acceleration in the first stage, and of the venous retardation of the second. To this state in which the sufferer exists, succeeds that of reaction. It is observable, in all the diseases to which man is liable, that there is an attempt of nature to produce reaction, causing those different stages in which the more acute diseases display themselves; in many of them there is a crisis, or that violent effort which seems to be the most determined struggle of the vis medicatrix for relief from the fearful state in which she has been thrown. During this, there is no organ which does not seem to be called into action for self-relief. This crisis more engaged the attention of the physicians of antiquity than those of the present day, as disease was allowed to take more of its own course, as well from the difficulty of finding those who were capable of treating it from its onset, as from the greater neglect of those habits of life now so thoroughly understood. During this crisis there is, as in ordinary insanity, a sudden change for the better where relief is obtained; but where it has not been watched and prepared for, there is a violent paroxysm, whose intensity bears proportion to the previous depression.

This action and reaction, so strongly marked in intermittent fever, affecting that portion of the nervous system which is contained within the vertebral column, producing the phenomena, in its greatest extent, of universal tremor, acting upon the brain, evidently causes a large portion of that which is displayed in the insane, under the guise of alternate depression and excitement. The sudden crisis which leads to impulsive acts in the insane that had previously exhibited the form only of imbecility and stupidity, most probably depends upon some altered state of the arterial circulation, some momentary determination to the brain; and, were we followers of the doctrines of Gall and of Spurzheim to their fullest extent, we should naturally be led to inquire whether the organ upon which they assert that homicide depends, was the seat of some instantaneous action. When we find that there exists in nature vegetable substances which produce this effect, or which suddenly call into action the desire for spilling blood, we must grant that, to some condition of the brain, produced by a physical agent, this is owing. It is not brandy alone that will in some constitutions cause a fearful ferocity, but there is a vegetable, a species of mushroom, called *arnanita muscaria*, whose effects are of the most striking character. Some of the Cossack tribes never go to battle without adding a portion to the spirituous liquors which they take, and they become inspired with a blood-thirstiness which nothing can resist: even the *cannabis indica* has been known to inspire even a reflecting and humane individual with a desire for destruction; it often fills the mind with an impulse that cannot be resisted. On one occasion, when Dr. Moreau had himself taken it, by way of experiment, he piteously entreated that the window should be immediately shut, as he felt coming over him an irresistible propensity to throw himself out.

The frequent recurrence of the idea, such as is detailed by Esquirol, shows that there are occasional crises; one of these instances has been quoted by Dr. Prichard, in his "View of the different forms of insanity." A lad, aged twenty-one, always of sombre and surly disposition, had lost his father at the age of fourteen, and had never exhibited much affection towards his mother. At the age of eighteen his defection increased; he shunned society, yet worked industriously at a manufactory, neither displaying in words nor actions any signs of insanity, but declared that he felt a strong inclination to commit murder, that there were moments when he felt that he could feel pleasure in killing his mother. When the horrible nature of these suggestions was set before him, and the punishments due to such an act, he exclaimed, "I am no longer master of myself." On several occasions, after having embraced his mother, his face became red, his eyes sparkled, and he cried out, "Mother, take care of yourself—I am forced to kill you." He very soon became calm, and shed tears. One day he met in the street a Swiss soldier, who was quite a stranger to him, seized his sword, and made a sudden effort to take it from him and stab him. On another occasion he drew his mother into the cellar, and attempted to kill her with a bottle. For six months this youth was agitated by this horrible impulse; he slept little, complained of his head, kept himself in solitude, and was insensible to the grief of his family, but exhibited no sign of mental aberration in his discourse. When brought to Charenton, he admitted with *sang froid* that he had been five or six times on the point of killing his mother and sister, and that he bore them no ill will, and had no fixed ideas. After ten months passed under Esquirol's care, this youth became perfectly recovered, and afterwards continued to be an affectionate son and an intelligent and industrious manufacturer.

As the individual who has a fixed idea, which remains known only to himself, makes no complaint, nor reveals the internal struggles which harass and destroy him, it is only when the madness bursts out with uncontrollable vehemence that it is known. The suicide has long fought within himself, before he has rushed to the fatal extreme; the homicidal monomaniac conceals for an immense length of time the horrors by which he is pursued, and it is only when at length he can bear his fate no longer, that he divulges his long-kept secret. The following case, which has been pronounced by one of our greatest authorities as the most curious exemplification of homicidal monomania, will be read with interest; it is a *procès verbal* of a French medical man, which will explain itself: "I, the undersigned, Guillaume Calmeilles, officier de santé, inhabiting and being domiciled in the chief town of Cazals, Lot, certify to those it may concern, that, upon the requisition of the mayor of the Commune of Marminiat, I went this day to the village of Brunet, in the said commune of Marminiat, to testify as to the state of mind of John Glenadel, a farmer, domiciled in the said village of Brunet. I found Glenadel seated upon his bed, having a cord round his neck fixed by one end to the foot of his bed; he had his arms fastened together at the wrists by another cord. To give my report faithfully, I do not think that I can do better than give the conversation which passed between us in the presence of his brother, his wife, and his sister-in-law. 'Are you ill?' 'I am very well—my health is almost too good.' 'What is your name?' 'John Glenadel.' 'What age are you?' 'Forty-three; I was born in '96—see if that is not correct.' 'Is it by force, or at your own wish, that you are thus tied up.' 'It is by my own consent, and indeed at my own wish.' 'Why is that?' 'To prevent me from the commission of a crime of which I have the utmost horror, and which I am impelled to commit in spite of myself.' 'What, then, is the crime?' 'I have an idea which attacks me, and relative to which I am no longer master of myself—I must kill my sister-in-law, and I shall do it if I am not prevented.' 'How long have you had that idea?' 'It is about six or seven years.' 'But have you anything to complain of your sister-in-law?' 'Not at all, sir; it is an unfortunate idea which I have, and I feel that I must carry it into execution.' 'Have you never had an idea of killing any other person than your sister-in-law?' 'I had formerly the thought of killing my mother; that took me when I was about the age of sixteen or seventeen, when I began to be a man, in 1812; I remember it very well. Since that time I have never had an hour's happiness, and I have been the most miserable of men.' 'You got over that first thought?' 'In 1822, I could no longer resist; I was then between twenty-five and twenty-six years of age. To take this unfortunate idea out of my head I entered the army as a substitute. I was two years in Spain with my regiment, then I returned to France, but this fixed idea followed me everywhere. More than once I was tempted to desert, that I might return to kill my mother. In 1826, I received an unlimited leave of absence, which I had not asked for, and I returned to my father's house; my dreadful idea returned with me. I passed four years with my mother, having an irresistible desire to destroy her.' What did you do, then?' 'Then, sir, seeing that I should infallibly commit a crime which alarmed me and filled me with horror, not to give way to the temptation I entered again as a substitute in the army. It was in the year 1830 that for the second time I left my father's roof; but the idea still pursued me, and at length I was decided to desert, to go and kill my mother.' 'You had, then, something to complain of with regard to your mother?' 'No, sir, I loved her very much; indeed, before leaving I said to myself, What! go and kill your mother, who so tenderly watched over your infancy, who loves you so much, in spite of all the melancholy idea which you nourish against her. No! never will I be guilty of it; but still you must kill some one. And then it was that the idea came into my head to kill my sister-in-law. I remember it very well—it was at Daix; it was in the year 1832. It was announced to me by mistake that my sister-in-law was dead; it was another of my relations who died. I then accepted the leave of absence which was given to me, but which I should not have done, had I not believed that she was no longer living; then, as soon as I arrived at home, and learnt that she was not dead, I felt a sudden seizure, a drag at my heart, which did me considerable injury, and my idea took its course.' 'What is the instrument with which you desire to kill your sister-in-law?' Here Glenadel melted away, his eyes were bathed in tears, he looked at his sister-in-law, and said, 'The instrument! the gentlest; but, whatever it is, once begun it must be finished; I know that I must see her dead—that is as certain as that God is God.' 'Are you not afraid to plunge your brother and your little nephews in misery and in despair?' 'That idea comes to me a little, but I shall be killed, and then I shall not see them. Such a monster as I am will be got rid of—I shall cease to live. I wish for no other happiness.' I then recollected that M. Grandsault de Salviat, my colleague and my friend, who is at the present moment in Paris, had spoken to me, about a year before, of a young man who, some years ago, had come to him, accompanied by his mother, to consult him upon a similar case, and, as such instances are exceedingly rare, I thought that it might have been Glenadel himself; I therefore asked him if it was he that had consulted my colleague, and he answered in the affirmative. 'What advice did M. Grandsault give you?' 'He gave me some excellent opinions, and afterwards bled me.' 'Were you at all relieved by the bleeding?' 'I did not find the least benefit from the bleeding; the idea still followed me with the same force.' 'I am about to give my report upon the state of your mind, and it will follow that you will be shut up in an asylum, where most probably you will be cured of your madness.' 'To cure me is impossible; but make your report as quickly as you can—that is of the greatest consequence. I cannot longer control myself.' 'Your parents must have given you good principles of morality, and good examples; and you must also have had an honest mind to have been enabled so long to resist so terrible a temptation.' Here Glenadel was again very much affected. He shed tears, and answered, 'Sir, you guess that; but that resistance is more painful than death. I see that I can no longer exist; I shall certainly kill my sister-in-law unless I am prevented,—that is as certain as God is God.' 'Glenadel,' I said, 'before I leave you, I request one favour: resist a few days longer; you will not much longer see your sister-in-law; we are about to remove you from hence, as you desire it so much.' 'Sir, I thank you; I will do my best to obey your recommendations.' I left the house, and as I went to mount my horse to take my departure, Glenadel desired that I should be called back; and, on my return, he said, 'Tell the gentlemen that I beg of them to place me where there is not the slightest chance of my escape, for I shall make every attempt to do so; and if I can escape, be assured that immediately my sister-in-law will be dead. I shall only escape to kill her. I beg you to tell the gentlemen so.' I assured him that I would do so; but as I saw that he was in a state of high excitement, I asked him whether the cord that bound his arms was sufficiently tight, and if he did not feel that he had sufficient strength to release himself. He made an attempt, and said that he feared he had. 'But if I find you something which will keep your arms more strongly bound, will you accept it?' 'With gratitude, sir.' 'In that case, I will ask the brigadier of the gens-d'armes to lend me that which he makes use of to tie the hands of prisoners, and I will send it to you.' 'You will very much oblige me.' I purposed going frequently to Glenadel to assure myself of the state of his mind; but after the long and painful conversation which I had held with him, and after that which had been said to me by my colleague, M. Grandsault, and likewise after that which his brother and sister-in-law reported to me, who are deeply affected in consequence of the melancholy condition of Glenadel, without repeating my visits, I am perfectly convinced that John Glenadel is afflicted with delirious monomania, characterized in him by an irresistible propensity to murder,—a monomania by which Papavoine and others have been afflicted, fortunately but few in number. In faith of which, I hereunto affix my name, &c." Here, then, was a desire for murder existing for twenty-six years, during which period the individual was perfectly capable of following all his avocations, but was haunted by furies worse than those which rendered the being of Orestes wretched, in consequence of the death of his mother, Clytemnestra, considered by the ancients an example of the greatest endurance to which human nature could be submitted.

The following case is given as one of equal interest, showing that an individual may have for a considerable length of time a fixed idea, without any other intellectual disorder, and may preserve in the eyes of every one an appearance of the soundest state of mind:—Augusta Wilhelmina Strohm, of thirty years of age, without having given any previous sign of melancholy, and without any appreciable motive, killed with a blow of a hatchet one of her friends, whom she had invited to see her. She went immediately and delivered herself up into the hands of the police. Whilst she was very young, Augusta Strohm had been present at the execution of a person of the name of Schaeffe, who was condemned to death for a murder. The care that had been taken to prepare this woman for death, her walk to the scaffold, had produced such an impression upon Augusta, that from that moment she looked upon that mode of termination of existence as a blessing,—that is to say, to be prepared for death, and to make such a religious end as the condemned. This thought never left her; but her moral principles for a long time carried on a struggle within her, until within about six weeks of the time when she murdered her friend. A man of the name of Kultafen was executed at Dresden. This second execution, by the circumstances with which it was accompanied, made a still stronger impression upon her mind, and served still further to excite her to carry out the first idea, and to urge her on to murder. Strohm was in her earliest youth when this first impression was made upon her imagination, and fifteen years elapsed before she carried into effect her latent madness. All that time she showed no extraordinary symptoms, but appeared perfectly rational, and no one had the slightest idea that there was within her any struggles. This may be ascribed to that love of imitation which exists in so extraordinary and unaccountable a degree in females, which is exhibited in so many various diseases—in hysteria, in epilepsy, in convulsions, and in every nervous state. When Harriet Cornier's case was the subject of so much interest, numerous similar examples occurred. Esquirol was frequently consulted. One lady heard a voice commanding her to kill her eldest boy. For a month she was pursued by an internal voice and an unceasing desire to strangle him. So frequently was she harassed by this fearful state, that she determined on and attempted suicide. A lady who read in a newspaper the condemnation of a criminal was so overcome by the impression that she saw before her constantly a bloody head, separated from the trunk, covered with a black crape. She experienced such a dreadful sensation from the ghastly sight which perpetually haunted her, that she made several attempts at suicide. Such cases, however, are more to be referred to diseases of imagination than impulsive insanity; they are rather hallucinations, dependent upon diseased emotions, than that irresistible feeling urging on to the commission of crime independent of any exciting cause. It is to be borne in mind that all those who have fixed ideas do not destroy themselves or injure others; they bear their maladies without any betrayal of the sad feeling which possesses them, and carry their silent sorrows to the grave. Disease will sometimes give origin to the fixed idea in those who previously were apparently in a sound state; but if their history be duly inquired into, it will be found that madness has been at some time or other developed in some branch of the family, and that it partakes of the nature of hereditary taint. Typhus fever has been known to generate this state, where it may have been previously latent; it is like a dream, and the idea is somewhat similar to one which remains after a dream has passed away. With regard to that monomania which is exhibited in females after childbirth, and which consists in a desire to destroy their offspring, that demands an investigation which has as yet been little thought of. That cerebral fever which terminates in partial delirium has doubtless often been the cause of child-murder, for which the unfortunate mother has been condemned to death; whilst, as far as regards the culpability which springs from consciousness of crime, and the power of distinguishing between right and wrong, she was innocent. These questions are yet to be agitated for the benefit of society, and to relieve the judgment-seat from the condemnation of those whose madness is their only crime.

